# Identification of the Linear Fc-Binding Site on the Bovine IgG1 Fc Receptor (boFcγRIII) Using Synthetic Peptides

**DOI:** 10.3390/vetsci11010024

**Published:** 2024-01-08

**Authors:** Ruining Wang, Junqing Guo, Ge Li, Xun Wang, Jifei Yang, Qingmei Li, Gaiping Zhang

**Affiliations:** 1 Key Laboratory of Animal Immunology, Henan Academy of Agricultural Sciences, Zhengzhou 450002, China; 28wangruining@163.com (R.W.); guojunqing@hnagri.org.cn (J.G.); yangjifei@hnagri.org.cn (J.Y.); 2 College of Veterinary Medicine, Henan University of Animal Husbandry and Economics, Zhengzhou 450046, China; 3 College of Veterinary Medicine, Northwest A&F University, Xianyang 712100, China; leegehz@126.com; 4 College of Veterinary Medicine, Henan Agricultural University, Zhengzhou 450046, China; wangxun0512@126.com; 5 Jiangsu Co-Innovation Center for Prevention and Control of Important Animal Infectious Disease and Zoonose, Yangzhou University, Yangzhou 225009, China

**Keywords:** BoFcγRIII, Fc-binding site, bovine IgG1, synthetic peptides

## Abstract

**Simple Summary:**

Bovine IgG1 Fc receptor (boFcγRIII) is a homologue to human FcγRIII (CD16) that has two extracellular Ig-like domains and can bind bovine IgG1 with medium–low affinity. We identified the Fc-binding site as well as its key residues for IgG1 binding using synthetic peptides, which is located in the second extracellular domain of boFcγRIII. It provides new insights for the IgG–Fcγ interaction and FcγR-targeting drugs development.

**Abstract:**

The bovine IgG1 Fc receptor (boFcγRIII) is a homologue to human FcγRIII (CD16) that binds bovine IgGI with medium–low affinity. In order to identify the Fc-binding site on the bovine IgG1 Fc receptor (boFcγRIII), peptides derived from the second extracellular domain (EC2) of boFcγRIII were synthesized and conjugated with the carrier protein. With a Dot-blot assay, the ability of the peptides to bind bovine IgG1 was determined, and the IgG1-binding peptide was also identified via truncation and mutation. The minimal peptide AQRVVN corresponding to the sequence 98–103 of boFcγRIII bound bovine IgG1 in Dot-blot, suggesting that it represents a linear ligand-binding site located in the putative A–B loop of the boFcγRIII EC2 domain. Mutation analysis of the peptide showed that the residues of Ala^98^, Gln^99^, Val^101^, Val^102^ and Asn^103^ within the Fc-binding site are critical for IgG1 binding on boFcγRIII. The functional peptide identified in this paper is of great value to the IgG–Fc interaction study and FcR-targeting drug development.

## 1. Introduction

Fcγ receptors (FcγRs) are expressed on the surface of effector leukocytes and have the ability to specifically recognize the Fc domain of IgG mediating various immunomodulatory events, with great influence on diverse aspects of innate and adaptive immunity [1]. It plays vital roles in humoral and cellular immune responses through interactions with the Fc region of immunoglobulin G (IgG), making those attractive targets for the development of novel immunotherapeutic strategies to autoimmune, infectious or malignant diseases as well as passive immunity [2,3,4,5]. Three different classes of FcγRI (CD64), FcγRIIA/B/C (CD32) and FcγRIIIA/B (CD16) have been extensively characterized in humans, while the fourth type of FcγRIV was identified in mice [6]. Among them, FcγRIII (CD16) is a medium–low-affinity IgG receptor whose extracellular region is similar to FcγRII and also contains two Ig-like domains. Human FcγRIII is encoded by homologous huFcγRIIIA and huFcγRIIIB genes. The extracellular regions of huFcγRIIIA and huFcγRIIIB are highly similar, but the transmembrane and intracellular regions are quite different. Human FcγRIIIA is a transmembrane receptor that binds human IgG1 and IgG3 monomers or complexes, but not IgG2 or IgG4. In contrast, FcγRIIIB has no transmembrane region and intracellular region, but is anchored to the cell membrane by glycosylphosphatidylinositol (GPI), which is effective for human IgG1 and IgG3 binding [7,8]. To date, several structures of the extracellular domain of huFcγRIII in complex with IgG1-Fc showed that the EC2 domain of the low-affinity FcγRIII is docked into the horseshoe opening of a homodimeric Fc region on IgG1, providing a novel strategy to design small peptides for regulating the interaction of IgG1 to FcγRIII [9,10,11,12,13,14]. It has actually been reported that the peptides of human IgG are able to bind with FcγR molecules [15,16,17]. Therefore, it is proposed that the Fc-binding peptides of the low-affinity FcγR can provide a novel strategy to regulate the FcR-mediated inflammatory responses.

The FcγRIII molecules have been identified and cloned in cattle and sheep, of which the boFcγRIII gene was first identified from the γ/δ T cell [18,19,20]. The full-length cDNA of boFcγRIII (1071 nt) contains a single open reading frame (ORF) of 750 nt, encoding a 250 aa receptor protein. The extracellular region is composed of 191 aa, of which four Cys are distributed at positions 47, 89, 128 and 172, forming two Ig-like domains. In addition, a variant gene of boFcγRIIIA was cloned from the cDNA library of bovine alveolar macrophages. The cDNA contains a single ORF (504 nt) encoding a 168 aa receptor protein, of which only two Cys distributed at the 31 and 74 positions formed a single Ig-like domain. However, the interaction between the receptor and IgG has not been reported yet.

In this study, synthetic peptide analysis was used to identify the bovine IgG-binding region of the receptor, and the linear ligand-binding site of boFcγRIII was initially identified with the aim to lay the foundation for the study of boFcγRIII–IgG interaction.

## 2. Materials and Methods

### 2.1. Purification of Bovine IgG1 and IgG2

Bovine IgG1 and IgG2 were isolated from the bovine anti-chicken red blood cells (RBCs) sera as described [21]. Briefly, bovine IgG was purified from the sera of calves immunized with RBCs using 18%, 16% and 14% of sodium sulphate precipitation after dialysis against 0.01 mol/L phosphate buffer (PB, pH 5.4). The purified bovine IgG was then applied to DEAE ion exchange chromatography to isolate IgG1 and IgG2, which was further purified by protein A Sepharose affinity chromatography, in which bovine IgG2 but not IgG1 was captured specifically by protein A. After digestion using a Pierce Fab Micro Preparation Kit (Thermo Scientific, Rockford, IL, USA), the Fab and Fc fragments of bovine IgG1 were isolated via protein G affinity chromatography (Supplementary Figure S1). The agglutination doses of IgG1 and IgG2 for RBC sensitization were titrated using 0.5% of chicken RBCs.

### 2.2. HRP Labeling of the Bovine IgG

The purified bovine IgG was coupled to horseradish peroxidase (HRP) through the modified sodium periodate method and used for peptide binding [22]. Briefly, 10 mg of the purified bovine IgG1 or IgG2 was incubated with 2 mg HRP (RZ > 3.0, Sigma-Aldrich Corp., St. Louis, MO, USA), which was pretreated with 0.06 mol/L NaIO_4_ and 0.16 mol/L ethylene glycol, followed by dialysis with 0.05 mol/L carbonate buffer (pH 9.5) at 4 °C overnight. After adding NaBH_4_ solution (5 mg/mL), the HRP-labeled bovine IgG1 or IgG2 was precipitated using 50% saturated ammonium sulfate and dialyzed against PBS overnight. The labeling efficiency and protein concentration of the HRP conjugates were determined by their OD_280_ and OD_403_ values.

### 2.3. Design and Synthesis of boFcγRIII Peptides

After alignment of the EC2 domain protein sequences of boFcγRIIIA (X99695) with moFcγRIII (NP_034318), huFcγRIIIA (NP_000560) and huFcγRIIIB (NP_000561), six peptides with a length of 11–16 amino acids derived from the sequence 97–171 of boFcγRIII were designed based on the huFcγRIIIB crystal structure [9,10]. The designed peptides were synthesized and characterized by GL Biochem Ltd. (Shanghai, China) using the solid-phase peptide synthesis technology [23]. An additional cysteine residue was added to the N-terminus of peptides that do not possess any cysteine residue for further conjugation. The synthetic peptides were then conjugated to bovine serum albumin (BSA) to improve their solubility and reactivity.

### 2.4. Conjugation of the boFcγRIII Peptides

With Sulfo-SMCC (Pierce Biotechnology Inc., Rockford, IL, USA) as a hetero-bifunctional cross-linker, the boFcγRIII peptides were conjugated to an IgG-free BSA (Sigma, St. Louis, MO, USA) to improve peptide reactivity according to the manufacturer’s instructions (Supplementary Figure S2). Briefly, 1 mg Sulfo-SMCC (MW: 436.37, Spacer arm length: 11.6 Å) dissolved in 50 µL DMSO was mixed with 4 mg IgG-free BSA in 0.5 mL coupling buffer (0.1 mol/L PB pH 7.2, 0.15 mol/L NaCl, 1 mol/L EDTA) at room temperature for 1 h, followed by dialysis against the coupling buffer overnight at 4 °C. Twenty microliters of peptide (100 µg) in 0.01 mol/L PB (pH 7.2) containing 5 mol/L EDTA and 12.5% (*v*/*v*) dimethylformamide (DMF) were then incubated with equal volume of the SMCC-activated BSA carrier protein (100 µg) at room temperature for 4 h and at 4 °C overnight (Figure S3). The concentration of the conjugated peptide was adjusted to 1 mg/mL using 0.01 mol/L PB (pH 7.2) for bovine IgG binding.

### 2.5. Peptide IgG-Binding Assay

Due to the poor solubility of the peptides, a Dot-blot assay was established to detect binding of the different boFcγRIII peptides to bovine IgG1. The boFcγRIII peptides coupled with BSA were spotted at 1 µg/dot, respectively, on a nitrocellulose membrane (Millipore, Bedford, MA, USA). After being air-dried, the membrane containing the peptide was incubated with 0.1% gelatin solution at 37 °C for 1 h to block the unspecific binding sites. The treated membrane was then added to the HRP-conjugated IgG1 or IgG2 (HRP-IgG1/IgG2) diluted at 10 µg/mL in the 0.1% gelatin solution, and incubated at 37 °C for 1 h. After being washed thoroughly with PBST, the membrane was colored using 3-amino-9-ethylcarbazole (AEC) (Sino-America Biotechnology Company, Luoyang, China) as a substrate. The colored dots on the membranes were then scanned under a TSR-3000 Strip Reader (Bio-Dot, Irvine, CA, USA), and the relative optical density (ROD) of the colored dots was analyzed using AIS analysis software (Ver. 6.0, Bio-Dot, Irvine, CA, USA).

### 2.6. Bovine IgG-Blocking Assay

In the bovine IgG-blocking experiment, the boFcγRIII peptides which conjugated with BSA (1 µg/dot) were spotted on the nitrocellulose membranes using BSA and recombinant protein as controls, respectively. After being blocked by 0.1% gelatin, the peptide-blotted membrane was incubated with the Fab and Fc fragments (10 µg/mL), bovine IgG1 (10 µg/mL), and PBS, respectively, at 37 °C for 1 h. The preincubated membranes were further detected by HRP-IgG1 and colored using the AEC staining kit as described above.

### 2.7. Truncation and Mutation of the Fc-Binding Peptide

The amino acid residues were first deleted one by one from the N-terminus of the Fc-binding peptide of boFcγRIII, resulting in a series of the N-truncated peptide. The residues at its C-terminus were then deleted one by one from the N-truncated peptide with Fc-binding ability to determine the shortest peptide with the ability of binding bovine IgG1. Furthermore, the residues within the minimal peptide with Fc-binding activity were replaced with Ala amino acid, individually generating the Ala-substituted peptides to identify the key residues of the Fc-binding site for bovine IgG1. The derivatives of the Fc-binding peptide, including the N-truncated, C-truncated and Ala-mutated peptides, were synthesized and coupled to BSA for Dot-blot detection as described above.

### 2.8. Model Building of boFcγRIII

Since the crystal structures of the extracellular domains of FcγRIII show remarkable similarity, the primary amino acid sequence of boFcγRIIIA (X99695) was submitted to the SWISS-MODEL Protein Structure Homology Modelling Server (https://swissmodel.expasy.org/interactive#structure, accessed on 17 May 2023), and the crystal structure of human IgG1 Fc fragment–FcγRIII complex (PDB DOI: 1E4K) was used to model the target sequence in its oligomeric form with ProMod3 [24]. The conserved coordinates between the target and the template were submitted to the model. Insertions and deletions were remodeled using a fragment library. Side chains were then rebuilt. Finally, the geometry of the resulting model was regularized by using a force field. The structural characteristic of the Fc-binding site was then analyzed based on the boFcγRIII structure model.

## 3. Results

### 3.1. Bovine IgG1 Binding to the boFcγRIII Peptides

**Figure 1:**
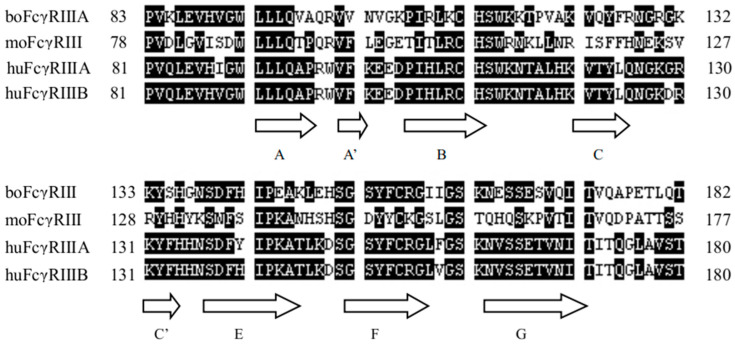
Alignment of the protein sequences of the EC2 domains of boFcγRIIIA (X99695), moFcγRIII (NP_034318), huFcγRIIIA (NP_000560) and huFcγRIIIB (NP_000561). The black highlights show that three or four sequences are the same, and the dotted lines represent deletions of amino acids. β-Sheets of the huFcγRIIIA EC2 domain (named from A to G) are shown by blank arrows below the sequence according to previous report [9].

**Figure 2:**
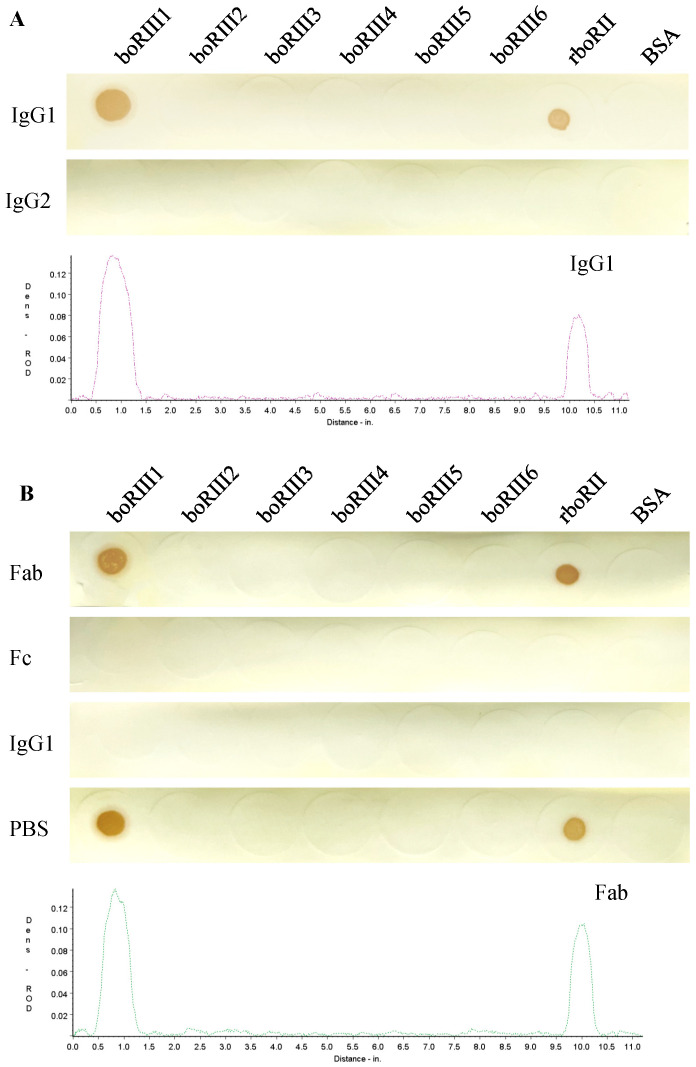
Bovine IgG1 binding to the boFcγRIII peptides. The boFcγRIII peptides (1 µg/dot) spotted on the nitrocellulose membranes were tested with the HRP-IgG1 at 37 °C for 1 h, followed by AEC color development using the recombinant boFcγRII (rboRII) and BSA as the positive control and negative control, respectively (**A**). To determine the specificity of Fc-binding, the peptide membrane was incubated with the Fab and Fc fragments (10 µg/mL), purified bovine IgG1 (10 µg/mL) and PBS, respectively, at 37 °C for 1 h, followed by HRP-IgG1 detection (**B**). The colored membranes were scanned under a TSR-3000 Reader, and relative optical density (ROD) values were analyzed using AIS software.

In order to determine Fc-binding sites on the boFcγRIII, six peptides with a length of 11–16 amino acids (Table 1), which were derived from the A–B, B–C, C–C’, D–E, E–F and F–G putative loops of the EC2 domain, respectively, were designed and synthesized after the EC2 domain sequence alignment of boFcγRIII, huFcγRIIIA, huFcγRIIIB and moFcγRIII (Figure 1). The binding activity of HRP-IgG1 or HRP-IgG2 to the boFcγRIII peptides was determined using the Dot-blot test. The results showed that the HRP-IgG1 bound specifically to the first peptide boRIII1 of ^97^VAQRVVNVGKPIRLK^111^ showing an obviously colored dot, which is located at the putative A–B loop of the boFcγRIII EC2 domain. Meanwhile, HRP-IgG1 showed no binding to the other boFcγRIII peptides because no colored dot was found for the peptides of boRIII2 to boRIII6 (Figure 2A). In addition, HRP-IgG2 binding to any of the six boFcγRIII peptides was not found in the Dot-blot assay, indicating that boFcγRIII binds bovine IgG1 specifically but not bovine IgG2. In the blocking assay, the binding of HRP-IgG1 to the boRIII1 peptide was completely blocked by the Fc fragment of IgG1, whereas the Fab fragment had no inhibitory effect on the binding of HRP-IgG1, indicating that it does not bind the peptide of boFcγRIII (Figure 2B).

### 3.2. Determination of the Fc-Binding Site for Bovine IgG1

**Figure 3:**
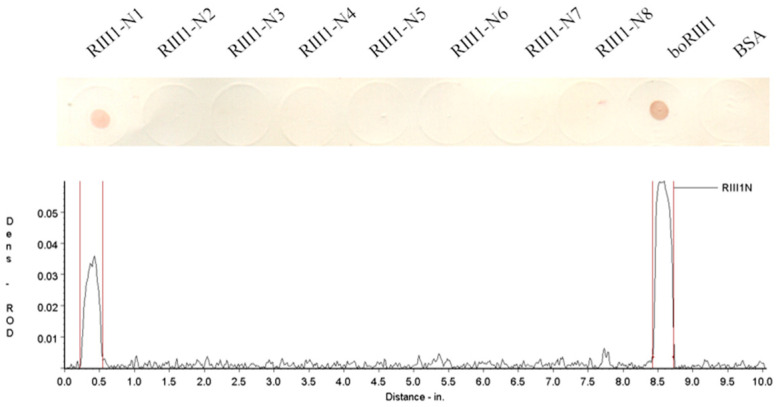
IgG1 binding of the N-truncated boRIII1 peptides. The N-truncated peptides derived from boRIII1 spotted on the nitrocellulose membranes were tested with the HRP-IgG1 as described above. The colored membranes were then scanned under the TSR-3000 Strip Reader, and the ROD values were obtained.

**Figure 4:**
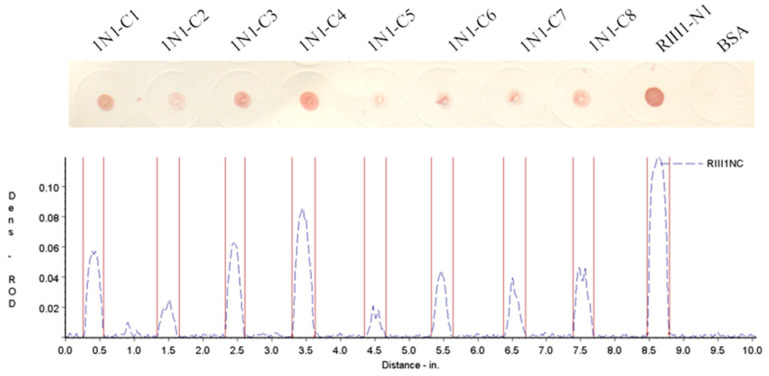
IgG1 binding of the C-truncated boRIII1 peptides. The N-truncated peptides derived from boRIII1 spotted on the nitrocellulose membranes were tested with the HRP-IgG1 as described above. The colored membranes were then scanned under the TSR-3000 Strip Reader, and the ROD values were obtained.

The boRIII1 peptide that binds bovine IgG1 specifically was then truncated from N-terminus and C-terminus to determine the shortest peptide with binding ability of bovine IgG1. The amino acid residues were first deleted one by one from the N-terminus of boRIII1 peptide, and the eight N-truncated peptides (RIII1-N1 to N8) were then determined using the Dot-blot test for binding bovine IgG1 (Table 2). The Dot-blot results showed that the deletion of Val^97^ residue from the N-terminal of boRIII1 did not significantly weaken the binding of bovine IgG1, but further deletions completely lost the Fc-binding ability of the peptide (Figure 3). The peptide RIII1-N1 that was able to bind bovine IgG1 was then used to further delete amino acid residues from the C-terminus, and another eight C-truncated peptides (1N1-C1 to C8) were produced (Table 3). Dot-blot results showed that all eight C-truncated peptides maintained their binding ability to bovine IgG1 until the Asn^103^ residue was deleted (Figure 4). Therefore, the peptide truncation results showed that the eight-residue peptide ^98^AQRVVN^103^ is the shortest peptide with binding ability for bovine IgG1 and is considered as the Fc-binding site on boFcγRIII at the putative A–B loop of the boFcγRIII EC2 domain.

### 3.3. Crucial Residues for Bovine IgG1 in the Fc-Binding Site

**Figure 5:**
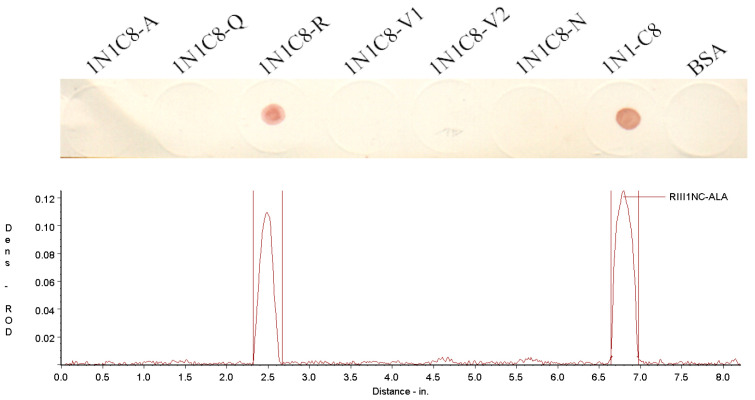
IgG1 binding of the Ala-substituted 1N1-C8 peptides. The Ala-substituted peptides derived from 1N1-C8 dotted on the nitrocellulose membranes were tested with the HRP-IgG1 as described above. The colored membranes were then scanned under the TSR-3000 Strip Reader, and the ROD values were obtained.

To determine the key residues for binding bovine IgG1 in the Fc-binding site, the residues of the Fc-binding peptide 1N1-C8 were replaced with alanine one by one (Table 4) and tested with Dot-blot assay. The results indicated that the mutation of Ala^98^, Gln^99^, Val^101^, Val^102^ or Asn^103^ with Ala (except for Ala^98^ with Gly) completely abolished IgG1-binding ability of the peptide 1N1-C8, whereas the mutation of Arg^100^ to Ala did not weaken the binding of bovine IgG1 (Figure 5), suggesting that the Ala^98^, Gln^99^, Val^101^, Val^102^ and Asn^103^ residues of the Fc-binding site are the key residues for binding bovine IgG1 on boFcγRIII.

### 3.4. Structural Characteristic of the Fc-Binding Site on boFcγRIII

**Figure 6:**
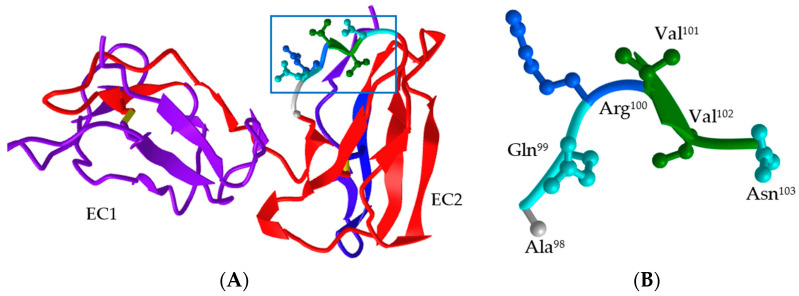
Structure model of the extracellular domains of boFcγRIII. The structure model of boFcγRIII in monomer was built based on the human IgG1 Fc fragment–FcγRIII complex crystal structure (PDB DOI: 1E4K) with ProMod3 on the SWISS-MODEL Server, in which the Fc-binding site located on the first loop of the EC2 domain was shown in box (**A**), and the residues with side chains were labeled (**B**).

Since the crystal structure of boFcγRIII is not obtained, its structure model in monomer was built based on the human IgG1 Fc fragment–FcγRIII complex crystal structure (PDB DOI: 1E4K) with ProMod3 on the SWISS-MODEL Server (Figure 6A). The sequence alignment showed that the sequence identity of boFcγRIIIA (X99695) and huFcγRIIIB (NP_000561) was 62.72%, indicating that its structure could be predicted using homology-modeling. The global and per-residue model quality was assessed using the QMEAN scoring function, in which the GMQE (global model quality estimate) value and QMEANDisCo global score were calculated as 0.55 and 0.73 ± 0.07, respectively. In the built structure model, boFcγRIII consists of two immunoglobulin (Ig)-like EC domains, similar to those of FcγRII and FcγRIII. The EC1 domain contains 79 amino acid residues (10–88) forming 1 α-helix of H1 (65–67) and 7 putative β-sheets of S1 (10–15), S2 (20–21), S3 (27–32), S4 (43–46), S5 (57–60), S6 (69–74) and S7 (84–88), while the EC2 domain has 83 residues (92–174) forming 2 α-helixes of H2 (114–117) and H3 (148–150) and 9 putative β-sheets of S8 (92–96), S9 (101–102), S10 (108–113), S11 (121–127), S12 (130–137), S13 (141–143), S14 (152–160), S15 (163–169) and S16 (170–174). There are several flexible loops between the secondary structures with different lengths linking the α-helixes and β-sheets. The Fc-binding site of ^98^AQRVVN^103^ was located at the first loop and S9 sheet of the EC2 domain of boFcγRIII. The side chains of the six residues formed a flexible loop and a small sheet of S9 was found between two Val residues (Figure 6B).

## 4. Discussion

Previously, the boFcγRIII gene was cloned from the cDNA library of bovine alveolar macrophages, which encodes a receptor protein with a single extracellular domain possessing an 82 aa deletion in the EC2 domain [18]. When the cDNA was used to transfect COS-7 cells, there was no rosette formation found on the transfected cells. First, the FcR γ chain or TCR ζ chain is essential for the expression of FcγRIIIA on the cell surface, and FcγRIIIA cannot be expressed on the cell surface unless both the FcR γ chain and TCR ζ chain are cotransfected. Therefore, COS-7 transfected with boFcγRIII alone could not express receptor molecules on the cell surface due to the absence of the FcR γ chain. Second, the protein encoded by the mutant gene lacks the IgG-binding region of EC2, and even if it is expressed on the cell surface, it cannot combine with IgG-RBC to form a rosette. Therefore, the complete boFcγRIIIA and bovine FcR γ chain genes would be cloned into the corresponding eukaryotic expression plasmids and followed by cotransfection of both cDNA to establish a functional research platform for boFcγRIII.

FcγRIII interacts with the lower hinge/upper CH2 of IgG in a 1:1 stoichiometry fashion, and its IgG-binding site is also located in the EC2 domain. Mutation analysis shows that the C–C’ loop and F–G loop of the EC2 region are the main IgG-binding sites [25,26]. The crystal structure of the IgG1 Fc fragment–huFcγRIII complex showed that the residues of the receptor EC2 region and the EC1–EC2 junction region bind with the Fc fragment Cγ2 and the low-hinge region through ionic bonds, hydrogen bonds and hydrophobic interactions. The Fc-binding region of FcγRIII includes Trp^110^-Ala^114^ located at the B–C loop, Val^155^-Lys^158^ at the F–G loop, His^116^-Thr^119^ at the C-strand and Asp^126^-His^132^ at the C’-strand of the EC2 domain. In addition, the residues of Gly^129^, Arg^152^ and Ile^85^-Trp^87^ are also involved in the IgG binding [27,28]. Furthermore, not only the amino acid, but also the glycan composition can greatly influence the affinity of FcγRIII for the Fc [29,30]. In this study, the 98–103 polypeptide AQRVVN of boFcγRIII showed good binding activity, which has been applied for patent protection in China (application no. CN202310670273.6). The linear ligand-binding site is located in the A–B loop of the EC2 domain of the receptor, while the B–C loop (boRIII2), C–C’ loop of the EC2 region (boRIII3) and F–G loop (boRIII6) polypeptides did not have obvious binding reaction with HRP-IgG1, which was significantly different from the ligand binding site of human FcγRIII. Because the peptide scanning method could not identify the conformational binding sites on boFcγRIII, further mutation studies on boFcγRIII need to be performed to analyze the key amino acid residues in its EC2 domain involved in the binding of bovine IgG1.

Recent studies have proven that in mouse natural killer cells, the receptors of CD16 and CD32b Fcγ are involved in regulating the antibody-mediated responses [31]. The Fc-FcγRIII engagement and alveolar macrophages are required for vaccine-induced antibody-mediated protection against antigen-shifted variants of SARS-CoV-2 as well as primary amebic meningoencephalitis in mouse models [32,33]. In a systemic lupus erythematosus (SLE) mouse model, the MRL/lpr mice treated with the Fc-binding peptide of huFcγRII showed the increased survival and reduce renal injury [34]. Therefore, the Fc-binding site peptide modulating the interaction of FcγR and Fc should provide an alternative strategy for the development of drugs to regulate antibody-based inflammation. In 2020, a molecular docking method based on the crystal structure of a receptor or protein was developed for designing a peptide ligand, which was first used for the purification of a virus antigen [35]. Since then, several designed affinity peptides have been used for the precise assembly of multiple antigens on nanoparticles [36]. In addition, the virtual screening-based affinity peptides were also used to inhibit virus infection such as hepatitis B and D viruses (HBV/HDV) [37]. Although the crystal structure of boFcγRIII or the Fc-receptor complex has not been resolved yet, the structure model of boFcγRIII was built by homology modeling using the human IgG1 Fc fragment–FcγRIII complex crystal structure as a template in this study. Moreover, the structure model of bovine IgG1 can be built using the SWISS-MODEL Server, which would provide structural support to understand the molecular basis of the interaction between the Fc-binding site and bovine IgG1. Furthermore, the Fc-binding peptide of boFcγRIII could be further designed or mutated using the molecular docking method based on the structure model to improve its binding affinity to bovine IgG1, showing great potential for the development of FcR-targeting drugs.

## 5. Conclusions

This study identified the linear Fc-binding site located in the putative A–B loop of the EC2 domain on boFcγRIII using synthetic peptides, in which the residues of Ala^98^, Gln^99^, Val^101^, Val^102^ and Asn^103^ are critical for IgG1 binding on boFcγRIII. It provides new insights for the IgG–Fcγ interaction and FcγR-targeting drugs development.

## Figures and Tables

**Table 1:** Characteristics of the boFcγRIII peptides.

Name	Sequence	Length (aa)	Mass (Da)	Isoelectric Point	IgG1 Binding ^b^	Predicted Position ^c^
boRIII1	^97^(C) ^a^ VAQRVVNVGKPIRLK^111^	16	1780.14	11.66	+	A–B loop
boRIII2	^112^CHSWKKTPVAKV^123^	12	1383.62	10.58	−	B–C loop
boRIII3	^124^(C)QYFRNGRGKKYS^135^	13	1606.77	10.58	−	C–C’ loop
boRIII4	^136^(C)HGNSDFHIPE^145^	11	1255.28	5.07	−	D–E loop
boRIII5	^146^(C)AKLEHSGSYF^155^	11	1241.33	7.15	−	E–F loop
boRIII6	^156^CRGIIGSKNESSESVQ^171^	16	1693.77	6.45	−	F–G loop

^a^ The residue of Cys was added to the N-terminus for the peptide containing no Cys residues for BSA conjugation. ^b^ The bovine IgG1 binding to the boFcγRIII peptides was determined via Dot-blot assay. “+” means positive for bovine IgG1 binding, and “−” means negative for bovine IgG1 binding. ^c^ The putative position was named based on the crystal structure of huFcγRIII.

**Table 2:** N-terminal truncations of the boRIII1 peptide.

Name	Sequence	Length (aa)	Mass (Da)	Isoelectric Point	IgG1 Binding ^b^
RIII1-N1	^98^(C) ^a^ AQRVVNVGKPIRLK^111^	15	1681.01	11.66	+
RIII1-N2	^99^(C)QRVVNVGKPIRLK^111^	14	1609.94	11.66	−
RIII1-N3	^100^(C)RVVNVGKPIRLK^111^	13	1481.81	11.66	−
RIII1-N4	^101^(C)VVNVGKPIRLK^111^	12	1325.63	10.89	−
RIII1-N5	^102^(C)VNVGKPIRLK^111^	11	1226.50	10.89	−
RIII1-N6	^103^(C)NVGKPIRLK^111^	10	1127.37	10.89	−
RIII1-N7	^104^(C)VGKPIRLK^111^	9	1013.27	10.89	−
RIII1-N8	^105^(C)GKPIRLK^111^	8	914.14	10.89	−

^a^ The residue of Cys was added to the N-terminus for the peptide containing no Cys residues for BSA conjugation. ^b^ The bovine IgG1 binding to the boFcγRIII peptides was determined via Dot-blot assay. “+” means positive for bovine IgG1 binding, and “−” means negative for bovine IgG1 binding.

**Table 3:** C-terminal truncations of the RIII1-N1 peptide.

Name	Sequence	Length (aa)	Mass (Da)	Isoelectric Point	IgG1 Binding ^b^
1N1-C1	^98^(C) ^a^ AQRVVNVGKPIRL^110^	14	1552.84	11.59	+
1N1-C2	^98^(C)AQRVVNVGKPIR^109^	13	1439.69	11.59	+
1N1-C3	^98^(C)AQRVVNVGKPI^108^	12	1283.51	10.64	+
1N1-C4	^98^(C)AQRVVNVGKP^107^	11	1170.36	10.64	+
1N1-C5	^98^(C)AQRVVNVGK^106^	10	1073.25	10.64	+
1N1-C6	^98^(C)AQRVVNVG^105^	9	945.08	9.55	+
1N1-C7	^98^(C)AQRVVNV^104^	8	888.03	9.55	+
1N1-C8	^98^(C)AQRVVN^103^	7	788.90	9.55	+

^a^ The residue of Cys was added to the N-terminus for the peptide containing no Cys residues for BSA conjugation. ^b^ The bovine IgG1 binding to the boFcγRIII peptides was determined via Dot-blot assay. “+” means positive for bovine IgG1 binding, and “−” means negative for bovine IgG1 binding.

**Table 4:** Ala substitutions of the 1N1-C8 peptide.

Name	Sequence	Length (aa)	Mass (Da)	Isoelectric Point	IgG1 Binding ^b^
1N1C8-A	^98^(C) ^a^ GQRVVN^103^	7	774.88	9.55	−
1N1C8-Q	^98^(C)AARVVN^103^	7	731.84	9.55	−
1N1C8-R	^98^(C)AQAVVN^103^	7	703.79	5.12	+
1N1C8-V1	^98^(C)AQRAVN^103^	7	760.84	9.55	−
1N1C8-V2	^98^(C)AQRVAN^103^	7	760.84	9.55	−
1N1C8-N	^98^(C)AQRVVA^103^	7	745.87	9.55	−

^a^ The residue of Cys was added to the N-terminus for the peptide containing no Cys residues for BSA conjugation. ^b^ The bovine IgG1 binding to the boFcγRIII peptides was determined via Dot-blot assay. “+” means positive for bovine IgG1 binding, and “−” means negative for bovine IgG1 binding.

## Data Availability

Data are contained within the article and Supplementary Materials.

## Supplementary Materials

The following supporting information can be downloaded at: https://www.mdpi.com/article/10.3390/vetsci11010024/s1, Figure S1: SDS-PAGE analysis of the purified bovine IgG1, as well as its Fab and Fc fragments; Figure S2: Two-step reaction scheme for conjugating peptide and BSA with Sulfo-SMCC; Figure S3: SDS-PAGE analysis of the boFcγRIII peptides coupled with BSA.
