# Supplementary material for: Identification of the Linear Fc-Binding Site on the Bovine IgG1 Fc Receptor (boFcγRIII) Using Synthetic Peptides
Source: Vet Sci. 2024 Jan 8;11(1):24. doi: 10.3390/vetsci11010024 (PMC10818675; doi:10.3390/vetsci11010024)
Supplement: Supplementary file 1 [file vetsci-11-00024-s001.zip › Model Building Report.pdf]

SWISS-MODEL Homology Modelling Report

Model Building Report

This document lists the results for the homology modelling project "bovine Fc gamma receptor III" submitted to SWISS-MODEL workspace on May 17, 2023, 2:24 a.m..The submitted primary amino acid sequence is given in Table T1.

If you use any results in your research, please cite the relevant publications:

- Waterhouse, A., Bertoni, M., Bienert, S., Studer, G., Tauriello, G., Gumienny, R., Heer, F.T., de Beer, T.A.P., Rempfer, C., Bordoli, L., Lepore, R., Schwede, T. SWISS-MODEL: homology modelling of protein structures and complexes. Nucleic Acids Res. 46(W1), W296-W303 (2018).
- Bienert, S., Waterhouse, A., de Beer, T.A.P., Tauriello, G., Studer, G., Bordoli, L., Schwede, T. The SWISS-MODEL Repository - new features and functionality. Nucleic Acids Res. 45, D313-D319 (2017).
- Studer, G., Tauriello, G., Bienert, S., Biasini, M., Johner, N., Schwede, T. ProMod3 - A versatile homology modelling toolbox. PLOS Comp. Biol. 17(1), e1008667 (2021).
- Studer, G., Rempfer, C., Waterhouse, A.M., Gumienny, G., Haas, J., Schwede, T. QMEANDisCo - distance constraints applied on model quality estimation. Bioinformatics 36, 1765-1771 (2020).
- Bertoni, M., Kiefer, F., Biasini, M., Bordoli, L., Schwede, T. Modeling protein quaternary structure of homo- and hetero-oligomers beyond binary interactions by homology. Scientific Reports 7 (2017).

Results

The user uploaded a template structure to use for the modelling process.

Models

The following model was built (see Materials and Methods "Model Building"):

Model #01	File	Built with	Oligo-State	Ligands	GMQE	QMEANDisCo Global
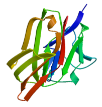	PDB	ProMod3 3.3.0	monomer	None	0.55	0.73 ± 0.07

Template	Seq Identity	Oligo-state	QSQE	Found by	Method	Resolution	Seq Similarity	Range	Coverage	Description
template_upload.1.C	62.72	monomer	-	HHblits	Unknown	-	0.49	23 - 191	0.68	Polypeptide

Excluded ligands

Ligand Name.Number	Reason for Exclusion	Description
BMA.3	Binding site not conserved.	BMA
BMA.12	Binding site not conserved.	BMA
FUC.9	Not in contact with model.	FUC
FUL.18	Not in contact with model.	FUL
GAL.15	Binding site not conserved.	GAL
GLA.6	Binding site not conserved.	GLA
MAN.4	Not in contact with model.	MAN
MAN.7	Not in contact with model.	MAN
MAN.13	Binding site not conserved.	MAN
MAN.16	Not in contact with model.	MAN
NAG.1	Binding site not conserved.	NAG
NAG.2	Binding site not conserved.	NAG
NAG.5	Binding site not conserved.	NAG
NAG.8	Not in contact with model.	NAG

Ligand Name.Number	Reason for Exclusion	Description
NAG.10	Binding site not conserved.	NAG
NAG.11	Binding site not conserved.	NAG
NAG.14	Binding site not conserved.	NAG
NAG.17	Not in contact with model.	NAG

```
Target                 MWQLLPPAALPVLVSADTQTADPSKAVVLLDPQWNHVLTNDRVTLKCQGDYPVED
template_upload.1.C    ----------------------LPKAVVFLEPQWYSVLEKDSVTLKCQGAYSPED

Target                 NSTKWWHNGTLISSQTPSYFIADVKVQDSGEYKCQTGLSAPSDPVKLEVHVGWLL
template_upload.1.C    NSTQWFHNESLISSQASSYFIDAATVNDSGEYRCQTNLSTLSDPVQLEVHIGWLL

Target                 LQVAQRVVNVGKPIRLKCHSWKKTPVAKVQYFRNGRGKKYSHGNSDFHIPEAKLE
template_upload.1.C    LQAPRWVFKEEDPIHLRCHSWKNTALHKVTYLQNGKDRKYFHHNSDFHIPKATLK

Target                 HSGSYFCRGIIGSKNESSESVQITVQAPETLQTVSSFFPPWHQITFCLVMGVLFA
template_upload.1.C    DSGSYFCRGLVGSKNVSSETVNITIT-----------------------------

Target                 VDTGLYFSVRRHLQSSEEWRDGKVTWSKGP
template_upload.1.C    ------------------------------
```

Materials and Methods

User Template Alignment

The user entered their own target sequence together with an uploaded a template structure file in PDB format.

Model Building

Models are built based on the target-template alignment using ProMod3 (Studer et al.). Coordinates which are conserved between the target and the template are copied from the template to the model. Insertions and deletions are remodelled using a fragment library. Side chains are then rebuilt. Finally, the geometry of the resulting model is regularized by using a force field.

Model Quality Estimation

The global and per-residue model quality has been assessed using the QMEAN scoring function (Studer et al.).

Ligand Modelling

Ligands present in the template structure are transferred by homology to the model when the following criteria are met: (a) The ligands are annotated as biologically relevant in the template library, (b) the ligand is in contact with the model, (c) the ligand is not clashing with the protein, (d) the residues in contact with the ligand are conserved between the target and the template. If any of these four criteria is not satisfied, a certain ligand will not be included in the model. The model summary includes information on why and which ligand has not been included.

Oligomeric State Conservation

The quaternary structure annotation of the template is used to model the target sequence in its oligomeric form. The method (Bertoni et al.) is based on a supervised machine learning algorithm, Support Vector Machines (SVM), which combines interface conservation, structural clustering, and other template features to provide a quaternary structure quality estimate (QSQE). The QSQE score is a number between 0 and 1, reflecting the expected accuracy of the interchain contacts for a model built based a given alignment and template. Higher numbers indicate higher reliability. This complements the GMQE score which estimates the accuracy of the tertiary structure of the resulting model.

References

- **BLAST**
  Camacho, C., Coulouris, G., Avagyan, V., Ma, N., Papadopoulos, J., Bealer, K., Madden, T.L. BLAST+: architecture and applications. BMC Bioinformatics 10, 421-430 (2009). doi>

- **HHblits**
  Steinegger, M., Meier, M., Mirdita, M., Vöhringer, H., Haunsberger, S. J., Söding, J. HH-suite3 for fast remote homology detection and deep protein annotation. BMC Bioinformatics 20, 473 (2019).

Table T1:

Primary amino acid sequence for which templates were searched and models were built.

```
MWQLLPPAALPVLVSADTQTADPSKAVVLLDPQWNHVLTNDRVTLKCQGDYPVEDNSTKWWHNGTLISSQTPSYFIADVKVQDSGEYKCQTGLSAPSDPV
KLEVHVGWLLLQVAQRVVNVGKPIRLKCHSWKKTPVAKVQYFRNGRGKKYSHGNSDFHIPEAKLEHSGSYFCRGIIGSKNESSESVQITVQAPETLQTVS
SFFPPWHQITFCLVMGVLFAVDTGLYFSVRRHLQSSEEWRDGKVTWSKGP
```

Table T2:

Template	Seq Identity	Oligo-state	QSQE	Found by	Method	Resolution	Seq Similarity	Coverage	Description
template_upload.1.C	62.72	monomer	-	HHblits	Unknown	NA	0.49	0.68	Polypeptide
template_upload.1.C	63.31	monomer	-	BLAST	Unknown	NA	0.49	0.68	Polypeptide

The table above shows the top 2 filtered templates. A further 3 templates were found which were considered to be less suitable for modelling than the filtered list.
template_upload.1.A, template_upload.1.B, template_upload.1.C
