# Supplementary material for: Identification of the Linear Fc-Binding Site on the Bovine IgG1 Fc Receptor (boFcγRIII) Using Synthetic Peptides
Source: Vet Sci. 2024 Jan 8;11(1):24. doi: 10.3390/vetsci11010024 (PMC10818675; doi:10.3390/vetsci11010024)
Supplement: Supplementary file 1 [file vetsci-11-00024-s001.zip › Supplymentary Figures.pdf]

## Supplementary Figures

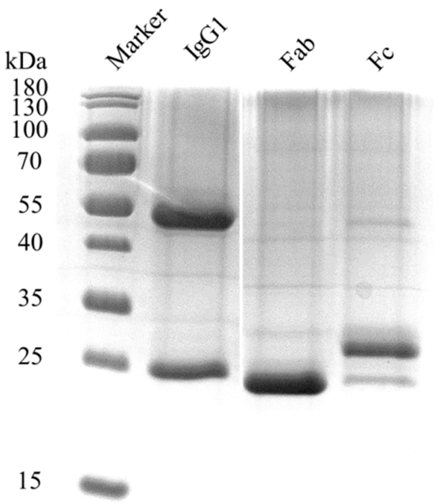


**Figure S1.** SDS-PAGE analysis of the purified bovine IgG1, as well as its Fab and Fc fragments.

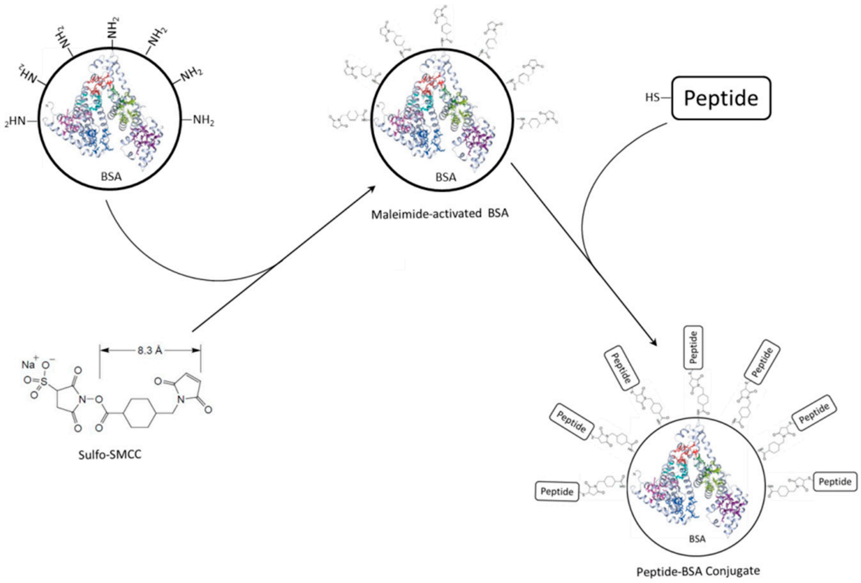


**Figure S2.** Two-step reaction scheme for conjugating peptide and BSA with Sulfo-SMCC.

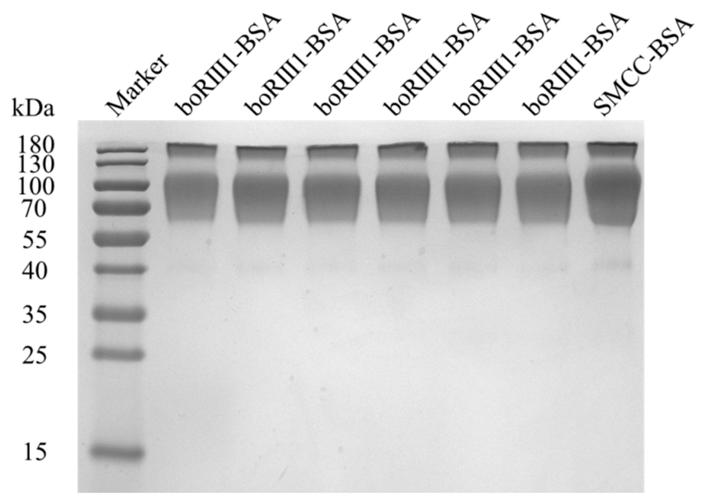


**Figure S3.** SDS-PAGE analysis of the boFcγRIII peptides coupled with BSA.
